# Metal Ion Release from Orthodontic Archwires: A Comparative Study of Biocompatibility and Corrosion Resistance

**DOI:** 10.3390/molecules29235685

**Published:** 2024-11-30

**Authors:** Marcin Mikulewicz, Piotr Suski, Oskar Tokarczuk, Magdalena Warzyńska-Maciejewska, Paweł Pohl, Beata Tokarczuk

**Affiliations:** 1 Department of Dentofacial Orthopaedics and Orthodontics, Division of Facial Abnormalities, Medical University of Wroclaw, Krakowska 26, 50-425 Wroclaw, Poland; marcin.mikulewicz@umw.edu.pl; 2 Dental Office “Corten Dental”, Makolągwy 21, 02-811 Warsaw, Poland; suskipiotr0@gmail.com; 3 Orthodontic Office “Ortomix”, Bursaki 25, 20-150 Lublin, Poland; 4 4th Military Clinical Hospital, 50-981 Wroclaw, Poland; mjwarzynska@gmail.com; 5 Department of Analytical Chemistry and Chemical Metallurgy, Faculty of Chemistry, University of Science and Technology, Wyspianskiego 27, 50-370 Wroclaw, Poland; pawel.pohl@pwr.edu.pl; 6 BT Orthodontic Office “Galeria Uśmiechów”, Polskie Towarzystwo Techniki Ortodontycznej, Plac Piłsudskiego 25, 51-152 Wroclaw, Poland; beatatokarczuk@gmail.com

**Keywords:** orthodontic wires, ion release, orthodontic allergy, NiTi wires, TMA, stainless steel

## Abstract

This study investigates the release of metal ions from commonly used orthodontic archwires, specifically, stainless steel (SS), nickel–titanium (NiTi), chromium–cobalt (CrCo), and titanium–molybdenum (TMA) alloys. To simulate oral conditions, each type of wire was immersed in artificial saliva at body temperature for a four-week period. Ion release levels were analyzed through ICP-OES mass spectrometry. The findings indicate that NiTi and CrCo wires released significantly higher amounts of nickel (Ni) and chromium (Cr) ions compared to SS wires. These findings underscore the potential risk of allergic reactions, particularly to nickel, and emphasize the need for careful consideration of biocompatibility in orthodontic material selection. This research also provides valuable insights aimed at minimizing adverse reactions in patients, especially those with metal allergies.

## 1. Introduction

Orthodontic archwires are essential components in dental treatment, generating the forces necessary to move teeth into their intended positions. These wires are typically made from various alloys, including stainless steel (SS), nickel–titanium (NiTi), chrome–cobalt (CrCo), and titanium–molybdenum (TMA) alloys, each offering distinct mechanical properties and biocompatibility benefits. Stainless steel, for instance, is widely used in orthodontics for its durability and corrosion resistance, while NiTi wires are valued for their shape memory and elasticity, qualities that are especially beneficial during the early stages of orthodontic treatment [1].

Nickel–titanium archwires, introduced in the 1960s by Andreasen, are characterized by high elasticity, shape memory, and resistance to permanent deformation [2]. NiTi wires demonstrate significant changes in mechanical behavior and applied force in response to temperature fluctuations, a feature relevant in both conventional and thermally activated NiTi wires. While exposure to high temperatures may lead to irreversible deformation, any temporary strain at lower temperatures can be reversed upon reheating [3]. Despite these advantages, the biocompatibility of NiTi wires is under scrutiny due to the allergenic, cytotoxic, and potentially mutagenic effects associated with nickel content [4].

Stainless steel has been used in orthodontics since the development of the Standard Edgewise technique and remains a popular choice in contemporary straight-wire methods. The alloy’s chromium content of 12–13% enables the formation of a thin, passivating layer of chromium oxide, which protects against corrosion by limiting oxygen diffusion into the deeper alloy layers [1]. Compared to nickel–titanium alternatives, stainless steel wires are less likely to provoke allergic reactions and are easily manipulated, allowing for welding and soldering connections. Although they offer a cost-effective option, stainless steel wires are relatively rigid and have limited flexibility, often necessitating more frequent adjustments during treatment [5].

Beta-titanium (β-Ti) wires, also known as TMA, were introduced for orthodontic use in 1979 by Goldberg and Burstone, who recognized their potential advantages for orthodontics. These wires offer an elastic modulus lower than that of stainless steel and close to the modulus of conventional NiTi alloys, along with excellent formability, weldability, and a low risk of hypersensitivity [6,7]. Since TMA wires do not contain allergenic nickel, they are considered one of the most biocompatible options and are recommended for patients prone to immune reactions triggered by allergens [1]. However, β-Ti wires present certain drawbacks, including a high surface roughness, which increases friction at the wire–bracket interface during sliding, and a susceptibility to fracture during bending [8]. To address surface roughness, nitrogen ion implantation has been used, though some studies have questioned its effectiveness in reducing friction [9,10]. TMA wires are commonly employed in the finishing phase of treatment, as well as for tooth rotation and in the formation of bending loops [1].

The advantages of chrome–cobalt alloys were recognized in the 1950s, leading to their adoption in orthodontics with the development of the Elgiloy archwire. Known for its flexibility, thermomechanical properties, and high corrosion resistance, Elgiloy quickly gained popularity [1]. It is available in various forms—Blue (soft), Yellow (elastic), Green (semi-elastic), and Red (flexible). The Blue variant is the most commonly used due to its formability and the potential to enhance its durability through heat treatment [11,12]. Today, Elgiloy is widely applied in palatal expanders [1].

Allergic reactions in orthodontics are often caused by immune hypersensitivity to metals like nickel, close to the conventional type IV (delayed-type) response. This reaction occurs in two phases: sensitization, where allergens (e.g., nickel ions) are recognized by immune cells and lead to the formation of memory T-cells, and elicitation, where re-exposure activates these T-cells, releasing inflammatory mediators such as cytokines. This results in localized inflammation, often manifesting as contact dermatitis with symptoms like erythema, swelling, or, in severe cases, oral ulceration. Allergic responses can also be triggered by ions from chromium, nickel, cobalt, copper, titanium, and silver [13]. Surface passivation layers—composed of chromium and titanium oxides—help slow ion release by reducing corrosion; however, these protective layers can degrade with mechanical wear, polishing, or lower pH levels [1]. Metal allergies are more common in women, especially following prior exposure to the allergen, as in cases of body piercing, where the immune system becomes sensitized to the metal [2].

Research on ion release from orthodontic archwires has expanded over the last decade, yet challenges persist in fully understanding the complex interplay between material properties, environmental conditions, and patient-specific factors. Our study contributes to this body of knowledge by providing insights into the role of material composition, environmental pH, and fluoride exposure in ion release dynamics, while emphasizing the clinical implications of these findings.

This study introduces a comprehensive analysis of ion release from orthodontic materials using advanced ICP-OES mass spectrometry, extending beyond nickel and chromium to include multiple ion types like copper and zinc. By systematically evaluating the effects of the material composition and wire shape and environmental factors such as pH and fluoride, it provides new insights into the dynamics of ion release under simulated oral conditions. Highlighting early-phase exposure risks and the role of emerging alloys like copper-enriched NiTi, this research offers practical guidelines for improving biocompatibility and patient safety in orthodontic treatment.

The aim of this study was to evaluate and compare the release of metal ions from various orthodontic archwires under simulated oral conditions. Specifically, it sought to quantify the release of nickel (Ni), chromium (Cr), and other metals from stainless steel, NiTi, TMA, and chrome–cobalt wires over a defined period in an artificial saliva environment.

## 2. Results

The results for ion release and its concentration in artificial saliva are presented in Table 1 including the content of each specific ion in each archwire used.

**Figure 1:**
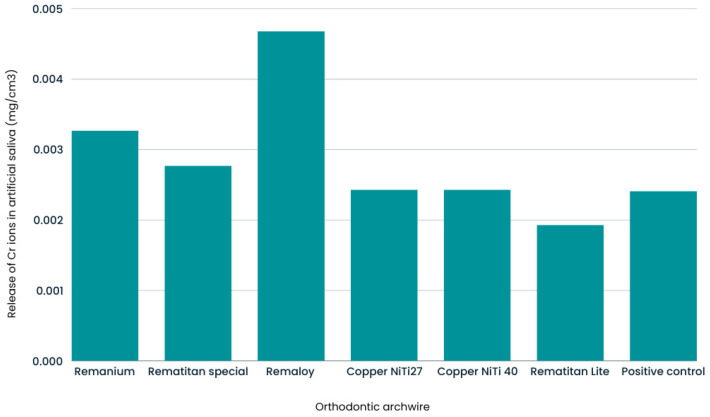
Mean Cr levels for six types of wire and the control group.

The test revealed that most variables significantly deviated from normal distribution, except for Co, Sn, and V, which showed approximately normal distributions. Due to the significant release of Ni and Cr, their prevalence in the alloy compositions and their known biological relevance and potential toxicity in dental applications were thoroughly investigated to determine whether their release was significantly affected by the type of wire used. One-way ANOVA was performed. The results showed statistically significant differences for Cr ions (F(6.47) = 17.28; *p* < 0.001; ω^2^ = 0.64) and Ni ions (F(6.47) = 22.53; *p* < 0.001; ω^2^ = 0.71), indicating that at least one wire group differed from the others. A post hoc analysis using the Games–Howell correction (for unequal variances) showed that the pure NiTi wire group (Rematitan Lite) had a significantly lower release of Cr ions compared to all other groups. The other groups did not show significant differences in Cr ion release. The mean values and 95% confidence intervals are presented in Table 2 and visualized in Figure 1.

Since the assumptions of homogeneity of variances and normality were violated, the results were confirmed using the non-parametric Kruskal–Wallis test. This test also indicated significant differences between the groups. Multiple Mann–Whitney tests (a non-parametric post hoc equivalent) showed more significant differences between groups, although the accumulation of Type I error must be considered. These tests revealed significant differences in all comparisons except for Rematitan special and CuNiTi (both variants had 27 and 40 degrees) and the control group.

**Figure 2:**
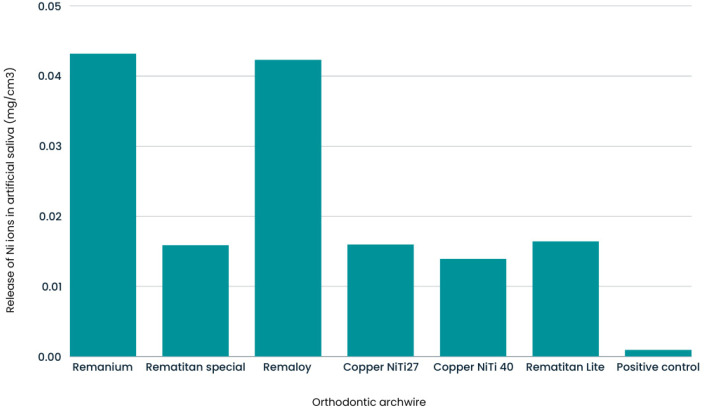
Mean Ni levels for six types of wire and the control group.

For Ni ion release, the post hoc analysis showed the highest result for the Remaloy wire, although it did not significantly differ from the Remanium wire. The Remanium wire had relatively high results but did not significantly differ from other wires. The control group had significantly lower results compared to all other groups except for Remanium and Rematitan. Similar findings were observed in the non-parametric tests, where Remaloy and Remanium wires showed higher results compared to others. The control group had the lowest results overall. The mean values are presented in Table 3, and a visual representation is shown in Figure 2.

## 3. Materials and Methods

### 3.1. Orthodontic Archwire Choice

The study utilized various types of orthodontic archwires from two manufacturers: Dentaurum (Ispringen, Germany) and Ormco (Glendora, CA, USA). From Dentaurum, the following archwires were used: Stainless Steel Remanium, Nickel–Chromium–Cobalt Remaloy, Nickel–Titanium Rematitan Lite, Titanium–Molybdenum Rematitan Special, and brass separation wire, which served as a positive control due to it having the most cytotoxic effects in the oral cavity out of all orthodontic materials used in clinical practice. From Ormco, copper–nickel–titanium thermal archwires (Copper NiTi) with activation temperatures of 27 °C and 40 °C were used. Each type of orthodontic archwire (Table 4) is described in detail, including the elemental composition and manufacturer specifications. The percentage content of the elements is presented in Table 5.

### 3.2. Artificial Saliva Environment

The experiments were conducted in an artificial saliva environment prepared according to Fusayama’s composition, with a pH of 6.75, simulating the physiological conditions of the oral cavity. The temperature of the test environment was maintained at 37 °C, and the artificial saliva contained NaCl, KCl, Na_2_S·9H_2_O, urea (CO(NH_2_)_2_), and NaH_2_PO_4_, which allowed for the replication of the natural properties of oral fluids. The composition of the artificial saliva is presented in Table 6. Highly pure reagents were used to prepare the artificial saliva (Table 7), which minimized contamination and ensured the reliability of the experimental results.

### 3.3. Incubation Procedure and Ion Release Analysis

One hundred and eight triangular samples of orthodontic arches with a side length of 1 cm were incubated in polypropylene containers filled with 30 mL Fusayama’s artificial saliva solution. The incubation period lasted for 4 weeks, with the temperature kept constant at 37 °C. After the incubation period, a quantitative analysis of the released metal ions was performed using ICP-OES mass spectrometry (Agilent, model 720). The analysis was conducted in two independent research centers at Wrocław University of Science and Technology: the Department of Advanced Materials Technology and the Department of Analytical Chemistry and Chemical Metallurgy, ensuring the repeatability of the results.

Potential sources of error included contamination from reagents, which could introduce impurities affecting the measurements. This was mitigated by using ultrapure reagents to prepare all solutions and samples.

### 3.4. Statistical Analysis

To address the research questions and test the proposed hypotheses, statistical analyses were conducted using the IBM SPSS Statistics 23.0 software package. Descriptive statistics were analyzed, including the Kolmogorov–Smirnov test with the Lilliefors correction, as well as a series of one-way analyses of variance. A significance level of *p* < 0.05 was adopted for this study. Results with significance levels between 0.05 < *p* < 0.09 were considered significant at the level of a statistical trend.

## 4. Discussion

Our results confirm that nickel and titanium ions are released at varying rates depending on the wire composition and shape and environmental conditions [14]. Notably, rectangular wires, as demonstrated by Azizi et al. [4], tend to release higher ion levels than round wires during initial exposure, a trend supported by our findings. This underscores the need for careful material selection during treatment planning, especially for patients with known sensitivities. Similarly, the accelerated ion release observed in acidic and fluoride-rich conditions aligns with findings from Espinoza-Montero et al. [15], Tahmasbi et al. [16], and Pastor et al. [17], highlighting the critical role of oral hygiene and dietary habits in modulating corrosion and ion release.

Artificial saliva, commonly used in simulated studies, provided a more stable environment for our experiments, reflecting real-world conditions and corroborating findings by Jamilian et al. [18]. However, as observed in our study and supported by Senkutvan et al. [19], pH fluctuations, particularly in acidic conditions, can significantly elevate nickel ion release. These results emphasize the importance of accounting for individual variability in oral environments, including factors such as saliva composition and fluoride exposure.

A key observation in our study was the influence of alloy composition on ion release. For instance, NiTi alloys released ions at a greater rate than stainless steel, particularly in acidic environments. These results are consistent with prior studies (e.g., Kuhta et al. [20], Metwally et al. [21], and Basting et al. [22]) that underscore the role of protective oxide layers in mitigating corrosion. Additionally, the incorporation of copper into NiTi alloys, as demonstrated by Titiz et al. [23], appears to enhance biocompatibility by reducing nickel release, a trend that we also observed in specific CuNiTi compositions.

Our findings further highlight the variability among modern orthodontic materials in minimizing ion release and their potential clinical implications. While ceramic brackets are often perceived as safer due to their reduced metal ion release [24,25], they may still contribute to overall exposure when paired with certain wires. This variability underscores the importance of tailoring material selection to patient-specific needs, as noted by Aiswareya et al. [24]. Furthermore, the risks associated with counterfeit or substandard materials, as described by Haleem et al. [26], reinforce the need for stringent quality control measures to ensure patient safety.

The temporal aspect of ion release, with nickel levels peaking within the first three months of treatment, as reported by Velasco-Ibanez et al. [27], aligns with our observations and suggests a need for close monitoring during this period. This is particularly critical for patients at risk of allergic reactions or systemic effects due to prolonged exposure.

This study focuses on a four-week observation period, offering insights into the early-phase ion release dynamics of orthodontic materials. However, ion release may evolve over time due to cumulative wear, oxide layer degradation, and oral environment changes. Research suggests that corrosion rates can stabilize or increase after several months, highlighting the need for extended observation to better understand long-term biocompatibility and implications for prolonged orthodontic treatment [28].

Overall, our study contributes to the growing understanding of ion release dynamics by emphasizing the interplay between material properties, environmental factors, and patient-specific variables. The findings provide valuable insights into the early-phase behavior of orthodontic materials, with direct clinical relevance. Understanding the variability in ion release across different alloys can guide material selection, particularly for patients with metal sensitivities. Further research should investigate these factors under more dynamic, real-world conditions to refine recommendations for patient management and improve the safety and efficacy of orthodontic materials.

This study has certain limitations that should be acknowledged. The use of artificial saliva as a model for the oral environment, while providing controlled conditions, does not fully replicate the complexity of the oral cavity, including mechanical stresses, dietary influences, and microbiological activity, all of which can affect ion release. Additionally, variations in pH [22,25] and exposure to fluoride [16,17,28], which occur naturally in clinical settings, may significantly influence corrosion dynamics and ion release rates.

## 5. Conclusions

Based on the research conducted, several key conclusions can be drawn. The study demonstrated that Remaloy and Remanium alloys release Cr ions at twice the rate of other wires, indicating that these materials have a higher susceptibility to corrosion, which may pose potential biocompatibility risks. Additionally, Remaloy and Remanium showed the highest release of Ni ions among the materials tested, which could elevate the risk of allergic reactions in patients with nickel sensitivity. The findings also revealed that wire geometry significantly influences the dynamics of metal ion release, with rectangular wires releasing more ions than round ones. Environmental factors, particularly the presence of fluoride in mouthwashes or an acidic pH, were found to exacerbate the corrosion of orthodontic wires, especially those made from NiTi, underscoring the need for careful material selection in such conditions.

Clinicians should prioritize nickel-free alloys, like TMA, for patients with metal allergies, and consider materials with lower ion release rates, such as copper-enriched NiTi. Researchers should focus on long-term studies under varied conditions to further improve material biocompatibility and patient safety.

Overall, the results highlight the importance of a thoughtful approach to selecting orthodontic materials, taking into account both alloy composition and corrosion characteristics, to reduce the risk of adverse reactions in patients and enhance the biocompatibility of orthodontic devices.

## Figures and Tables

**Table 1:** The number of ions released into artificial saliva—a comparison of the % ion content in the alloy and the results of ion release studies 1 and 2.

Ion	Archwire	Alloy Composition [%]	I Test [mg/cm^3^]	II Test [mg/cm^3^]
Fe	Remanium	70	0.0057	0.0602
	Rematitan special	-	0.0017	-
	Remaloy	4–6	0.0047	-
	Copper NiTi 27	-	-	-
	Brass	≤0.01	-	-
	Rematitan lite	≤0.5	-	-
	Copper NiTi 40	-	-	-
Cr	Remanium	18–20	0.0031	0.0045
	Rematitan special	-	0.0026	0.0026
	Remaloy	18–22	0.0042	0.0043
	Copper NiTi 27	0.2	0.0007	0.0024
	Brass	-	0.0019	0.0013
	Rematitan lite	≤0.5	-	0.0019
	Copper NiTi 40	0.2	0.0027	0.0024
Cu	Remanium	-	0.0025	-
	Rematitan special	-	-	-
	Remaloy	-	-	-
	Copper NiTi 27	5	0.0007	-
	Brass	62–65	3.2	2.3
	Rematitan lite	Significant	-	-
	Copper NiTi 40	5	0.0026	-
Mn	Remanium	≤2	0.0033	-
	Rematitan special	-	0.00105	-
	Remaloy	3–5	0.001	-
	Copper NiTi 27	-	0.0002	-
	Brass	≤0.1	-	-
	Rematitan lite	-	-	-
	Copper NiTi 40	-	0.0002	-
Ni	Remanium	8–10.5	0.0396	-
	Rematitan special	-	0.0145	-
	Remaloy	19–23	0.0435	-
	Copper NiTi 27	49.1	0.0133	-
	Brass	≤0.3	0.0167	-
	Rematitan lite	50–60	-	-
	Copper NiTi 40	49.1	0.0159	-
Pb	Remanium	-	<0.003	<0.003
	Rematitan special	-	-	-
	Remaloy	-	-	-
	Copper NiTi 27	-	-	-
	Brass	≤0.1	-	-
	Rematitan lite	-	-	-
	Copper NiTi 40	-	-	-
Ti	Remanium	-	0.0002	0.0002
	Rematitan special	78	0.0002	0.0003
	Remaloy	0.1–2	0.0002	0.0002
	Copper NiTi 27	45	0.0005	-
	Brass	-	0.0015	-
	Rematitan lite	45	-	0.0003
	Copper NiTi 40	45	-	0.0002
Zn	Remanium	-	0.003	-
	Rematitan special	-	0.0007	-
	Remaloy	-	0.001	-
	Copper NiTi 27	-	0.0006	-
	Brass	30–40	1.8	1.7
	Rematitan lite	-	-	-
	Copper NiTi 40	Significant	0.0007	-

**Table 2:** Average chromium (Cr) ion release based on the type of archwire used.

Ion Released	Archwire	N	Average	σ	95% Confidence		F	Significance	ω^2^
					↓ Limit	↑ Limit			
Cr	Remanium (SS)	6	0.00327	0.00057	0.00267	0.00387	17.28	17.28	0.64
	Rematitan Special (TMA)	6	0.00277	0.00042	0.00232	0.00321			
	Remaloy (CoCr)	6	0.00468	0.00149	0.00312	0.00625			
	Copper Niti 27	6	0.00243	0.00025	0.00217	0.00270			
	Copper Niti 40	6	0.00243	0.00005	0.00238	0.00249			
	Rematitan Lite (NiTi)	6	0.00193	0.00010	0.00182	0.00204			
	Positive Control	18	0.00241	0.00011	0.00235	0.00246			
	Summary	54	0.00275	0.00093	0.00249	0.00300			

σ—standard deviation.

**Table 3:** Average nickel (Ni) ion release based on the type of archwire used.

Ion Released	Archwire	N	Average	σ	95% Confidence		F	Significance	ω^2^
					↓ Limit	↑ Limit			
Ni	Remanium (SS)	6	0.04322	0.02385	0.01819	0.06824	22.53	22.53	0.71
	Rematitan Special (TMA)	6	0.01590	0.00991	0.00550	0.02630			
	Remaloy (CoCr)	6	0.04233	0.01269	0.02902	0.05565			
	Copper NiTi 27	6	0.01600	0.00412	0.01167	0.02033			
	Copper NiTi 40	6	0.01395	0.00385	0.00991	0.01799			
	Rematitan Lite (NiTi)	6	0.01645	0.00415	0.01209	0.02081			
	Positive Control	18	0.00097	0.00112	0.00041	0.00152			
	Summary	54	0.01675	0.01795	0.01185	0.02165			

σ—standard deviation.

**Table 4:** Orthodontic archwires used in research.

Type of Archwire	Producer
Orthos Copper NiTi 27 °C 0.018 upper/small (Part No: 219-0204) = NiTiCu 27 °C	Ormco Cooperation, (Glendora, CA, USA)
Orthos Copper NiTi 40 °C 0.016 × 0.022 upper/small (Part No: 219-5208) = NiTiCu 40°	Ormco Cooperation, (Glendora, CA, USA)
Rematitan special^®^ 0.016 × 0.022 Stan-gendraht, vierkant = TMA	Dentaurum, Ispringen, Germany
Remanium^®^ Stangendraht, vierkant, federhart, 0.41 × 0.56 mm/0.016 × 0.022 (REF: 766-602-00) = stainless steel	Dentaurum, GmbH Ispringen, Germany
Remaloy^®^ Stangendraht, vierkant, hart plus 0.41 × 0.56 mm/0.016 × 0.022 (REF: 537-510-00) = CoCr	Dentaurum, Ispringen, Germany
Rematitan^®^ Lite, 0.41 × 0.41 mm/0.016 × 0.016 (REF: 766-069-00) = pure NiTi	Dentaurum, Ispringen, Germany
Messing-Draht, Durchmesser 0.60 mm/23 (REF: 572-060-00) = pure brass	Dentaurum, Ispringen, Germany

**Table 5:** Percentage content of elements in the examined orthodontic archwires.

Archwire	Ni [%]	Cr [%]	Fe [%]	Mn [%]	Ti [%]	C [%]	Mo [%]	Si [%]	Cu [%]	Other [%]
CuNiTi	≤49.1	≤0.20	0	0	54	≤0.06	0	0	≤5.0	___
Rematitan Lite NiTi	50–60	0	≤0.5	0	39–49	≤0.1	0	0	0	H ≤ 0.1 N ≤ 0.1
Rematitian Special (TMA)	0	0	0	≤11.5	78	0	≤11.5	0	0	Zr ≤ 6 Sn ≤ 4.5
Remanium	6–9.5	16–18	68.2–73.9	2	0	≥0.15	≤0.8	≤2	0	*p* ≤ 0.0045 S ≤ 0.015 N ≤ 0.11
Remaloy (Co-Cr)	19–23	18–22	4–6	≤1.0	≥2.0	≤ 0.03	3–5	≤0.5	0	S ≤ 0.1 Co 35.4–49.5
Brass	≤0.3	0	≤0.01	≤0.1	0	0	0	0	62	Pb ≤ 0.1 Sn ≤ 0.1 Zn 36.9–37.4

**Table 6:** Composition of artificial saliva according to Fusayama.

Substance	Quantitative Composition [g/L]
NaCl	0.40
KCl	1.21
Na_2_S·9H_2_O	0.005
CO(NH_2_)_2_	1.00
NaH_2_ PO_4_	0.60

**Table 7:** Reagents used in the experimental section with artificial saliva.

Reagent	Formula	Purity	Brand	Origin Country	Producer
Natrium chloride	NaCl	99.99 Suprapur^®^	Merck	Germany	Gibco^®^/Life Technologies, Darmstadt
Potassium chloride	KCl	Trace metal basis ≥ 99.99%	Sigma-Aldrich	USA	Promega, Madison
Sodium sulfide nonahydrate	Na_2_S·9H_2_O	Trace metal basis ≥ 99.99%	Sigma-Aldrich	USA	Promega, Madison
Urea	CO(NH_2_)_2_	BioUltra for molecular biology ≥ 99.5%	Sigma-Aldrich	Germany	Biochrom, Berlin
Sodium dihydrogen phosphate	NaH_2_PO_4_	Trace SELECT^®^	Sigma-Aldrich	Germany	
Nickel(II) chloride hexahydrate	NiCl_2_·6H_2_O	Trace metal basis ≥ 99.999%	Sigma-Aldrich	USA	Promega, Madison
Human lysozyme, L1667			Sigma-Aldrich	USA	Promega, Madison
Nitric acid (V) 69%	HNO_3_	Tracepur^®^	Merck	Germany	

## Data Availability

The original contributions presented in the study are included in the article, further inquiries can be directed to the corresponding author.
